# Long-term outcomes of pseudomyxoma peritonei after cytoreductive surgery and hyperthermic intraperitoneal chemotherapy and its relevant risk factors in China: a retrospective study

**DOI:** 10.3389/fsurg.2026.1692847

**Published:** 2026-01-30

**Authors:** Shuncai Gao, Xiang Zhang, Ziyang Yu, Junwei Zhang

**Affiliations:** 1 Department of Anesthesiology, Aerospace Center Hospital, Beijing, China; 2 Department of Anesthesiology, Peking University School and Hospital of Stomatology, Beijing, China; 3 Department of Stomatology, Peking Union Medical College Hospital, Chinese Academy of Medical Sciences and Peking Union Medical College, Beijing, China; 4 Department of Anesthesiology, Changde Hospital, Xiangya School of Medicine, Central South University (The First people’s Hospital of Changde City), Changde, China

**Keywords:** chemotherapy, cox proportionalhazard models, cytoreductive surgery, evet-free survival, overall survival, pseudomyxoma peritonei, retrospective cohort study

## Abstract

**Objectives:**

Pseudomyxoma peritonei (PMP), generally spread of low grade appendiceal mucinous neoplasm (mucinous appendix neoplasms) into the abdominal cavity, is conventionally treated with cytoreductive surgery and hyperthermic intraperitoneal chemotherapy (CRS-HIPEC). Prognostic factors of small cohort sizes remain incomplete and conflicting. This large-scale study aimed to characterize long-term survival outcomes and identify prognostic factors in PMP patients following CRS-HIPEC.

**Materials and methods:**

We conducted a retrospective cohort study of 432 consecutive PMP patients treated with CRS-HIPEC at Aerospace Center Hospital (Beijing, China) from June 2014 to December 2020. Overall survival (OS) served as the primary endpoint, with event-free survival (EFS) as the secondary endpoint. Multivariable Cox proportional hazards models were employed to identify independent prognostic factors.

**Results:**

With median survival durations of 56 months (OS) and 45 months (EFS), cumulative mortality and event incidence reached 21.4% and 32.4%, respectively. Independent predictors for poorer OS included: preoperative raised tumor markers (hazard ratio [HR] = 4.90–10.20; 95% confidence interval [95% CI]: 1.11–46.67; *P* < 0.05), completeness of cytoreduction (CC) score (HR = 3.37–9.41; 95% CI: 1.05–16.37; *P* < 0.05), and high-grade PMP (HR = 1.80; 95% CI: 1.10, 2.93; *P* = 0.019). EFS was significantly associated with preoperative Barthel index (HR = 0.86; 95% CI: 0.74, 0.98; *P* = 0.019) in addition to the aforementioned factors. Intraoperative hypotension and hyperthermia were not associated with both OS and EFS.

**Conclusions:**

Key factors impacting outcomes of patients with PMP of mucinous appendix neoplasms included preoperative elevated tumor markers, Barthel index, CC-score, and the PMP histology, without intraoperative hypotension and hyperthermia.

## 1 Introduction

Pseudomyxoma peritonei (PMP) is a rare condition, with reported epidemiological variations across regions. In urban China, its estimated prevalence is 2.47 per million person-years, and the incidence is 1.19 per million person-years (1). In contrast, European data suggest a higher prevalence of 22 cases per million per year and an incidence of 3.2 per million per year (2). PMP is generally spread of low-grade mucinous appendix neoplasms into the abdominal cavity (3, 4). Currently, cytoreductive surgery combined with hyperthermic intraperitoneal chemotherapy (CRS-HIPEC) was endorsed by the Peritoneal Surface Oncology Group International as first-line therapy (5). Studies demonstrated that CRS-HIPEC at experienced centers with improved surgical techniques and integrated hemostasis techniques was safe (6) and could achieve 5- and 10-year survival rates of 74%–87% and 63%–73% in PMP patients, respectively (7, 8). Outcomes with CRS-HIPEC for PMP have been outstanding in prior studies because this disease is rather indolent with little metastatic and upstage potential. But, relapse after CRS-HIPEC was common, with the peritoneum being the most commonly relapse site, and the surgical resection of relapse disease could result in prolonged survival (9).

Prognostic factors for PMP after CRS-HIPEC remained incompletely characterized within limited studies except those concentrating on relapse of PMP (9) and perioperative safety after CRS-HIPEC (6). Key determinants included tumor-related factors (histologic grade, disease extent, peritoneal cancer index) (7, 10, 11), treatment-related parameters (completeness of cytoreduction, major postoperative complications, center experience) (12), and tumor markers (elevated CEA/CA199) (13). Current evidence showed conflicting results, with some studies emphasizing histologic grade alone while others highlighting multifactorial influences (7). Research progress is limited by PMP's rarity, resulting in small cohort sizes and unvalidated long-term outcomes (14).

Actually, aside from surgery-related factors, increasing studies demonstrated that anesthesia-related factors were also associated with surgical outcomes, as exemplified by intraoperative blood pressure management, especially hypotension (15–23). And in patients with cancer, evidence of relationship between intraoperative hypotension and long-term survival also had been proved (24–26). However, study reported the association between anesthesia-related factors and the long-term outcomes of PMP patients remained scarce.

Herein, this large-scale study aimed to investigate the long-term outcomes in PMP patients after CRS-HIPEC and the risk factors for long-term outcomes.

## 2 Materials and methods

### 2.1 Study design and patients

This retrospective cohort study included patients diagnosed with PMP confirmed by both surgical specialists and pathologists who underwent CRS-HIPEC at the Aerospace Center Hospital in Beijing, China, between June 2014 and December 2020. The study was approved by the Ethics Committee of the Aerospace Center Hospital (approval #2021-QT-014). Informed consent for the surgical specimens, imaging data, medical records used for academic exchange, scientific research, and teaching purposes was obtained from all patients and recorded in the Surgical Consent Form before all surgeries in our hospital in accordance with the local legislation and institutional requirements.

The inclusion criteria included patients aged ≥18 years and PMP originated from mucinous appendix neoplasms. The exclusion criteria included patients with missing intraoperative data, incomplete outcome data, repeated CRS-HIPEC procedure, or combined with intestinal contraction.

### 2.2 Perioperative procedures

All patients received standard intraoperative monitoring including electrocardiogram, invasive arterial blood pressure, pulse oximetry, end-tidal carbon dioxide, inhalational anesthetic concentration, nasopharyngeal temperature, and urine output measurement. General anesthesia was administered using a combination of propofol, volatile anesthetics (primarily sevoflurane), opioids, and muscle relaxants. Temperature management followed institutional protocols: employing an underbody warming blanket was used during CRS to prevent hypothermia (discontinued 30 min before HIPEC); while ice packs were applied to the head and neck region during HIPEC to mitigate systemic hyperthermia.

The CRS was performed according to Sugarbaker's technique (27), with surgical decisions made based on intraoperative findings. Following CRS, HIPEC was conducted using a closed-abdomen technique involving four catheters (two inflow/two outflow) connected to a perfusion system (RHL-2000B, Jilin Maida Technology Development Co., Ltd., China) that circulated chemotherapeutic agents (typically cisplatin 50–90 mg, 5-fluorouracil 1 g, and mitomycin C 10–40 mg) at 43.5 °C inflow/42.0 °C outflow temperatures, with perfusion rates of 600–1,000 mL/min for 60–90 min according to the Chinese Expert Consensus (28). After HIPEC, the abdomen was reopened for reconstruction and drainage. Postoperatively, selected patients received early postoperative intraperitoneal chemotherapy (EPIC, days 2–6) with 5-fluorouracil (1 g) ± cisplatin (40–100 mg) or raltitrexed (4–5 mg) per clinical judgment and guidelines.

### 2.3 Data collection

The preoperative data were gathered from a CRS-HIPEC database, encompassing demographic details (age, sex, and body mass index), overall health status [Charlson comorbidity index (29), American Society of Anesthesiologist classification (30), and Barthel index (31)], history of previous treatments [prior surgical score (PSS), prior chemotherapy, and HIPEC exposure], and preoperative laboratory findings (hemoglobin levels, and tumor markers including CA125, CA199, CEA).

The surgery-related data on the peritoneal cancer index (PCI) (32), the Completeness of Cytoreduction (CC) score (32), the duration of surgery, blood transfusion, estimated blood loss, and intraperitoneal chemotherapy regimens (chemotherapeutics of HIPEC and EPIC) were collected.

The postoperative information included tumor characteristics (origin, histology, and tumor markers), the incidence of acute kidney injury within 7 days (defined according to the KDIGO criteria) (33), and major complications within 30 days postoperative (classified as grade 3 or greater according to the Clavien-Dindo classification) (34). Histology of PMP was classified according to the 2016 Peritoneal Surface Oncology Group International consensus (35).

The intraoperative data were collected from the anesthesia information management system, including the volumes of artificial colloid and crystalloid infusion, mean arterial pressure (MAP), and core body temperature. Invasive MAP data were captured and stored at 30-seconds or 5-minutes intervals, and nasopharyngeal temperature data at a 5-min interval throughout the whole CRS-HIPEC procedure.

Postoperative follow-up information included postoperative computed tomography scans of the abdomen and pelvis), subsequent therapies for PMP, re-hospitalizations (with reasons and treatments), and patient survival status (with death dates confirmed against death certificates). The follow-up was censored at November 9, 2021. The data were from the medical charts including outpatient interviews or telephone interviews, following a schedule of every 3–6 months in the first 5 years post-surgery and then annually. For patients lost to follow-up, the date of the last hospital visit post-surgery was recorded as the censoring time.

### 2.4 Outcomes

The primary outcome was the overall survival (OS), defined as the interval from surgery to the date of death. The secondary outcome was event-free survival (EFS), defined as the interval from surgery to the date of PMP recurrence (for patients who achieved CC-0/1) or progression (for patients who achieved CC-2/3) (7), unplanned re-hospitalization for non-PMP diseases, or death, whichever occurred first. PMP recurrence/progression was defined as the reappearance/progression of the same local-regional peritoneal disease, on the evidence of computed tomography scans of the abdomen and pelvis.

### 2.5 Sample size

Owing to the rarity of this disease, the sample size was not calculated beforehand. We screened all the available patients during the study period in our center.

### 2.6 Statistical analysis

In order to reflect the fluctuation of MAP, the area under thresholds (AUT) of <MAP 75, 70, and 65 mmHg was calculated, respectively. The area under a specialized threshold was calculated by summarizing all areas (a_1_ + a_2_ + a_3_…) above the curve below the given thresholds. Each area was calculated according to the trapezoid rule and linear interpolation between adjacent measurements. As sensitive analyses, we also calculated the area above thresholds of nasopharyngeal temperature >38.0, 37.5, and 37 °C. All calculations were performed using the Python 3.10 software.

All analyses were performed using SPSS 27.0 (IBM SPSS, Armonk, NY, USA). The continuous data were presented as means ± standard deviations (SDs) for normally distributed data and as medians [interquartile ranges (IQRs)] for non-normally distributed data (according to the Kolmogorov–Smirnov test). The categorical data were presented as *n* (%). Pre-/perioperative variables, along with AUT of MAP, were explored for associations with OS and EFS using a univariable Cox proportional hazards regression model. The variables with *P* < 0.20 in the univariable analyses and AUT of MAP < 65 mmHg were included in a multivariable Cox proportional hazards regression model. Two-sided *P* values <0.05 were deemed statistically significant. Sensitivity analyses were performed after excluding patients missing intraoperative temperature data.

## 3 Results

**Figure 1:**
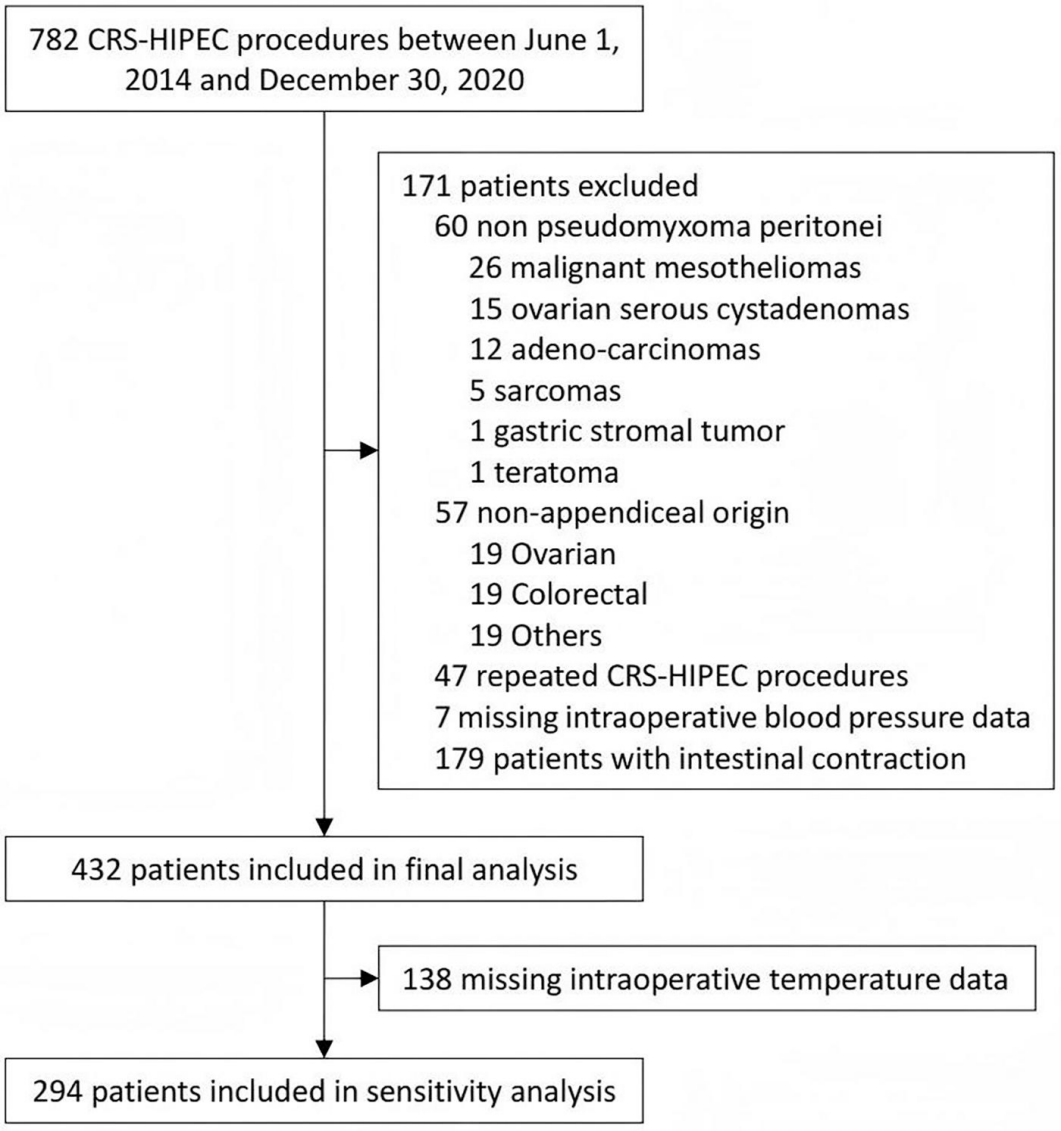
Study flowchart. CRS-HIPEC: combination of cytoreductive surgery and hyperthermic intraperitoneal chemotherapy.

The study screened 782 PMP patients undergoing CRS-HIPEC. Patients were excluded for the following reasons: 60 cases with non-confirmatory PMP diagnosis, 57 cases with non-appendiceal origins, 47 cases having multiple procedures, 7 cases missing MAP data, and 179 cases with intestinal contraction, leaving 432 patients for the final analyses (Figure 1).

In this cohort, 65.0% (281/432) were female, with mean age 57 ± 11 years (Table 1). Complete cytoreduction was achieved in 222 patients (51.4%), including 102 cases with CC-0 and 120 cases with CC-1. Intraperitoneal chemotherapy was administered as HIPEC alone (24.5%, *n* = 106) or HIPEC + EPIC (75.5%, *n* = 326). Intraoperative hypotension was ubiquitous: 431 (99.8%) patients experienced MAP < 75 mmHg, 427 (98.8%) patients MAP < 70 mmHg, and 405 (93.8%) patients MAP < 65 mmHg (Table 2 and Supplementary Table S5).

**Table 1:** Baseline data and their association with long-term survival (univariable COX regression analyses).

Characteristics	Statistical description (*n* = 432)	Overall survival		Event-free survival	
		Hazard ratio (95% CI)	*P* value	Hazard ratio (95% CI)	*P* value
Demographic characteristics					
Age, year	57 ± 11	1.02 (1.00, 1.04)	0.040	1.01 (0.99, 1.03)	0.243
Female sex	281 (65.0%)	1.11 (0.72, 1.69)	0.642	0.99 (0.70, 1.39)	0.931
Body mass index, kg/m^2^	23.2 (21.2, 26.0)	0.96 (0.90, 1.02)	0.191	1.00 (0.95, 1.05)	0.909
General status					
Charlson comorbidity index^a^	8.0 (8.0, 8.0)	0.92 (0.61, 1.39)	0.685	1.06 (0.79, 1.42)	0.702
ASA physical status^b^					
I-II	241 (55.8%)	Ref.		Ref.	
III-IV	191 (44.2%)	1.43 (0.95, 2.15)	0.086	1.32 (0.95, 1.85)	0.098
Barthel index^c^, per 10-point increase	10.0 (9.5, 10.0)	0.84 (0.73, 0.97)	0.017	0.84 (0.75, 0.95)	0.005
History of previous therapy					
Prior surgical score^d^					
0–2	312 (72.2%)	Ref.		Ref.	
3	120 (27.8%)	1.39 (0.90, 2.16)	0.143	1.40 (0.98, 2.00)	0.065
Prior chemotherapy	83 (19.2%)	1.44 (0.92, 2.26)	0.115	1.84 (1.28, 2.65)	0.001
Prior HIPEC exposure	88 (20.4%)	1.43 (0.87, 2.35)	0.162	1.42 (0.96, 2.12)	0.084
Preoperative laboratory tests					
Hemoglobin, g/L	117 ± 18 [14]	0.99 (0.97, 1.00)	0.011	0.99 (0.98, 1.0)	0.003
Albumin, g/L	37 ± 4 [10]	0.94 (0.90, 0.98)	0.007	0.92 (0.89, 0.96)	<0.001
Tumor markers (CA125, CA199, CEA)					
Normal	106 (24.5%)	Ref.		Ref.	
1 raised	113 (26.2%)	6.88 (1.63, 29.0)	0.009	4.17 (1.78, 9.85)	0.001
2 raised	98 (22.7%)	10.25 (2.41, 43.6)	0.002	6.04 (2.56, 14.28)	<0.001
All raised	115 (26.6%)	20.9 (5.05, 86.27)	<0.001	9.19 (3.95, 21.37)	<0.001

Data are mean ± SD, n (%), or median (interquartile range). Numbers in square brackets indicate patients with missing data. *P* values in bold indicate <0.20.

ASA, American society of anesthesiologists; HIPEC, hyperthermia intraperitoneal chemotherapy.

a Assessed according to Charlson comorbidity index (12 items) (30).

b Included ASA I (5 cases), ASA II (236 cases), ASA III (184 cases), and ASA IV (7 cases).

c Represented function capacity of patients, ranged from 0 (total dependence) to 100 (complete independence), assessed using the 10-item scale (each item was scored with 0, 5, 10 and 15 points) by nurse at hospital admission (32).

d Prior surgical score (PSS) ranged from 0 to 3. PSS-0 was for no prior surgery or biopsy; PSS-1 was for surgery in one abdominal region; PSS-2 was for surgery in 2–5 regions; PSS-3 was for surgery in >5 regions.

**Table 2:** Perioperative data and their association with long-term survival (univariable COX regression analyses).

Characteristics	Statistical description (*n* = 432)	Overall survival		Event-free survival	
		Hazard Ratio (95% CI)	*P* value	Hazard Ratio (95% CI)	*P* value
Intraoperative data					
Peritoneal cancer index^a^					
0–10	105 (24.3%)	Ref.		Ref.	
11–20	58 (13.4%)	4.59 (1.63, 12.90)	0.004	2.46 (1.22, 4.96)	0.012
21–30	153 (35.4%)	5.44 (2.17, 13.64)	<0.001	2.97 (1.67, 5.27)	<0.001
31–39	116 (26.9%)	5.71 (2.17, 15.01)	<0.001	2.85 (1.54, 5.28)	<0.001
Completeness of cytoreduction score^b^					
0	102 (23.6%)	Ref.		Ref.	
1	120 (27.8%)	4.79 (1.98, 11.57)	<0.001	2.36 (1.31, 4.25)	0.004
2	117 (27.1%)	4.41 (1.87, 10.42)	<0.001	2.51 (1.44, 4.37)	0.001
3	93 (21.5%)	5.09 (2.04, 12.68)	<0.001	2.71 (1.46, 5.02)	0.002
Complete cytoreduction^c^	222 (51.4%)	0.62 (0.41, 0.95)	0.028	0.65 (0.46, 0.92)	0.014
Duration of surgery, h	8.0 (6.5, 9.6)	1.08 (0.98, 1.19)	0.129	1.08 (1.00, 1.17)	0.063
Artificial colloid, per 500 mL	5.2 (4.0, 6.4)	1.16 (1.05, 1.29)	0.005	1.13 (1.04, 1.23)	0.005
Crystalloid, per 500 mL	6.8 (5.2, 8.4)	1.14 (1.03, 1.25)	0.008	1.09 (1.01, 1.18)	0.026
Blood transfusion	244 (56.5%)	2.67 (1.58, 4.52)	<0.001	1.97 (1.33, 2.90)	<0.001
Estimated blood loss, per 500 mL	3.0 (1.0, 3.2)	1.202 (1.09, 1.31)	<0.001	1.14 (1.06, 1.23)	<0.001
Area under threshold of MAP^d^, per 30 mmHg × min					
<75 mmHg	89 (45, 142)	1.00 (1.00, 1.01)	0.083	1.00 (1.00, 1.01)	0.056
<70 mmHg	43 (18, 80)	1.00 (1.00, 1.01)	0.075	1.00 (1.00, 1.01)	0.056
<65 mmHg	14 (4, 33)	1.00 (1.00, 1.01)	0.071	1.00 (1.00, 1.01)	0.077
Intraperitoneal chemotherapy regimens					
Intraperitoneal chemotherapy					
HIPEC alone	106 (24.5%)	Ref.		Ref.	
HIPEC plus EPIC ^e^	326 (75.5%)	0.49 (0.32, 0.76)	0.001	0.64 (0.45, 0.92)	0.017
Combinations of intraperitoneal chemotherapy-1					
Non-cisplatin containing	147 (34.0%)	Ref.		Ref.	
Cisplatin containing	285 (66.0%)	0.73 (0.46, 1.16)	0.178	0.76 (0.53, 1.08)	0.130
Combinations of intraperitoneal chemotherapy-2					
5-fluorouracil/mitomycin C^f^	147 (34.0%)	Ref.		Ref.	
Cisplatin only	75 (17.4%)	0.93 (0.49, 1.77)	0.833	0.81 (0.48, 1.37)	0.440
Cisplatin plus another^g^	173 (40.0%)	0.64 (0.37, 1.13)	0.122	0.74 (0.49, 1.11)	0.142
Cisplatin plus two others^h^	37 (8.6%)	0.49 (0.07, 3.61)	0.488	0.73 (0.23, 2.35)	0.600
Postoperative data					
Histopathology subtype					
Low-grade	323 (74.8%)	Ref.		Ref.	
High-grade	109 (25.2%)	2.61 (1.74, 3.92)	<0.001	1.81 (1.27, 2.56)	<0.001
Tumor markers (CA125, CA199, CEA)^i^					
Normal	198 (45.8%)	Ref.		Ref.	
1 raised	109 (25.2%)	1.58 (0.92, 2.73)	0.099	1.58 (1.02, 2.46)	0.040
≥2 raised^j^	125 (28.9%)	3.19 (1.94, 5.23)	<0.001	2.77 (1.86, 4.13)	<0.001
Acute kidney injury^k^	43 (10.0%)	1.26 (0.61, 2.60)	0.540	1.25 (0.70, 2.21)	0.452
Other major complications within 30 days^l^	93 (21.5%)	1.49 (0.94, 2.36)	0.087	1.30 (0.88, 1.91)	0.190

Data are *n* (%), or median (interquartile range). *P* values in bold indicate <0.20.

MAP, mean artery pressure; HIPEC, hyperthermia intraperitoneal chemotherapy; EPIC, early postoperative intraperitoneal chemotherapy.

a Peritoneal cancer index quantified peritoneal disease burden, and comprised a score of 0–3 in 13 abdominopelvic regions to a computed index ranging from 0 to 39. It was determined intraoperatively after abdominopelvic cavities exposure, but before any peritonectomy procedures performed (32).

b Completeness of cytoreduction (CC) score was used to record the volume of residual cancer, assessed after the surgical procedures. CC-0 signified no macroscopic residual disease remained; CC-1 signified no nodule greater than 2.5 mm in diameter remained; CC-2 signified nodule between 2.5 mm and 2.5 cm in diameter remained; and CC-3 signified nodule greater than 2.5 cm in diameter remained (32).

c A state of total macroscopic disease eradiation, encompasses CC-0 (no macroscopic residual disease remained) as well as CC-1 (no nodule greater than 2.5 mm in diameter remained) (32).

d Indicate areas under the specialized MAP thresholds, defined as sum of all areas below the given threshold, where each area was calculated with the use of trapezoid rule and linearly interpolating between measurements.

e Early postoperative intraperitoneal chemotherapy typically administered from postoperative days 2–6 as appropriate.

f Included 5-fluorouracil alone (4 cases), mitomycin C alone (1 case), and 5-fluorouracil + mitomycin C (142 cases).

g Included cisplatin plus 5-fluorouracil (171 cases), and cisplatin plus raltitrexed (2 case).

h Included cisplatin + 5-fluorouracil + raltitrexed (34 cases), cisplatin + 5-fluorouracil + mitomycin C (3 cases).

i Typically measured 14 days after surgery. If discharged within 14 days the last value was recorded.

j Included CA125 and CEA raised 15 cases, CA125 and CA199 raised 10 cases, CA199 and CEA raised 85 cases, all the three raised 15 cases.

k Defined either serum creatinine increased by ≥0.3 mg/dL within 48 h, or increased to ≥1.5 times baseline within the previous 7 days postoperative according to Kidney Disease Improving Global Outcome (KDIGO) criteria (33).

l Indicate Clavien-Dindo classification grade 3 or greater. Grade 3 indicated complications requiring radiological intervention, endoscopic, or surgical intervention with or without general anesthesia; grade 4 indicated complications requiring a return to the intensive care unit management, sepsis, and one or multiple organ failure; grade 5 indicated death within 30 days postoperative. Occurrence of acute kidney injury was not accounted for (34).

With 40-month median follow-up (IQR 25, 55), cumulative mortality was 21.8% (94/432) and event incidence was 32.4% (140/432). Readmissions for PMP occurred in 29.9% (129/432) vs. 57.2% (247/432) for planned interventions. Median survival durations were 56 (95% confidence interval [95% CI]: 50, 61) months for OS and 45 (95% CI: 40, 51) months for EFS (Table 3).

**Table 3:** Long-term follow-up results.

Characteristics	Statistical description (*n* = 432)
Duration of follow-up, month	40 (25, 55)
Re-hospitalization for PMP^a^	129 (29.9%)
Scheduled re-hospitalization^b^	247 (57.2%)
Number of deaths during follow-up	94 (21.8%)
Number of events during follow-up^c^	140 (32.4%)
Event-free survival, month^d^	45 (40, 51)
Overall survival, month (all-cause death)^e^	56 (50, 61)

Data are *n* (%), median (interquartile range), or median (95% confidence interval).

PMP, pseudomyxoma peritonei; HIPEC, hyperthermia intraperitoneal chemotherapy.

a Re-hospitalization for intravenous chemotherapy, HIPEC, or redo-cytoreduction surgery.

b Re-hospitalization for intravenous chemotherapy, HIPEC, regular follow-up visits, or removal of ureteral stents.

c Events refers to PMP recurrence (for patients achieved CC-0/1) or progression (for patients achieved CC-2/3), unplanned re-hospitalization for non-PMP serious diseases (included pyelostomy, inferior vena cava filter placement, endoscopic surgery, and surgery for hernia or other cancers), or all-cause death, whichever occurred.

d Defined as time interval from surgery to PMP recurrence/progression, unplanned re-hospitalization for non-PMP serious disease, or all-cause death, whichever occurred first.

e Defined as time interval from surgery to all-cause death.

Univariable analyses identified 24 candidate factors with *P* < 0.20 (Tables 1, 2) those associated with survival. After excluding factors that had correlation with others, 18 factors including AUT of MAP < 65 mmHg were included in the multivariable regression model. The multivariable analysis revealed that preoperative raised tumor markers (hazard ratio [HR] = 4.90–10.20; 95% CI: 1.11–46.67; *P* < 0.05), CC-score (HR = 3.37–9.41; 95% CI: 1.05–16.37; *P* < 0.05), and high-grade PMP (HR = 1.80; 95% CI: 1.10, 2.93; *P* = 0.019) were independently associated with OS. Similarly, the multivariable analysis identified that preoperative raised tumor markers (HR = 2.92–5.97; 95% CI: 1.15–16.07; *P* < 0.05), CC-score (HR = 1.87–4.24; 95% CI: 0.89–9.57; *P* < 0.05), and high-grade PMP (HR = 1.93; 95% CI: 1.15, 2.80; *P* = 0.022) were independently associated with EFS; otherwise, preoperative Barthel index (HR = 0.86; 95% CI: 0.74, 0.98; *P* = 0.019) was found to be associated with EFS (Table 4). Intraoperative hypotension was not associated with both OS and EFS.

**Table 4:** Risk factors of overall and event-free survival (multivariable COX proportional hazard model)^a^.

Parameter	Overall survival		Event-free survival	
	Hazard ratio (95% CI)	*P* value	Hazard ratio (95% CI)	*P* value
Age, year	1.01 (0.99, 1.04)	0.267	1.00 (0.98, 1.01)	0.589
Body mass index, kg/m^2^	0.98 (0.92, 1.05)	0.621	1.03 (0.97, 1.08)	0.330
ASA physical status				
I-II	Ref.		Ref.	
III-IV	1.24 (0.76, 2.01)	0.395	1.07 (0.72, 1.59)	0.736
Barthel index^b^, per 10-point increase	0.97 (0.81, 1.16)	0.201	0.86 (0.74, 0.98)	**0**.**019**
Prior surgical score^c^				
0–2	Ref.		Ref.	
3	0.80 (0.47, 1.35)	0.397	0.96 (0.64, 1.43)	0.831
Prior chemotherapy	1.22 (0.71, 2.09)	0.474	1.40 (0.91, 2.14)	0.126
Prior HIPEC exposure	1.05 (0.59, 1.85)	0.873	1.13 (0.72, 1.78)	0.586
Preoperative hemoglobin, g/L	0.99 (0.98, 1.01)	0.482	1.00 (0.99, 1.01)	0.975
Preoperative albumin, g/L	1.01 (0.94, 1.08)	0.763	0.95 (0.90, 1.00)	0.071
Preoperative tumor markers (CA125, CA199, CEA)				
Normal	Ref.		Ref.	
1 raised	4.90 (1.11, 21.61)	**0**.**036**	2.92 (1.15, 7.41)	**0**.**024**
2 raised	5.67 (1.22, 26.42)	**0**.**027**	3.94 (1.48, 10.48)	**0**.**006**
All raised	10.20 (2.23, 46.67)	**0**.**003**	5.97 (2.22, 16.07)	**<0**.**001**
Completeness of cytoreduction score^d^				
0	Ref.		Ref.	
1	3.37 (1.05, 6.04)	**0**.**011**	1.87 (0.89, 2.40)	0.077
2	5.62 (2.15, 10.64)	**<0**.**001**	3.79 (1.54, 5.57)	**0**.**018**
3	9.41 (3.77, 16.37)	**<0**.**001**	4.24 (2.40, 9.57)	**<0**.**001**
Duration of surgery, h	0.98 (0.86, 1.13)	0.797	0.97 (0.87, 1.09)	0.626
Blood transfusion	1.09 (0.53, 2.24)	0.817	1.18 (0.67, 2.05)	0.572
Aera under MAP < 65^e^, per 30 mmHg × min	1.01 (0.99, 1.01)	0.770	1.00 (0.99, 1.01)	0.883
Intraperitoneal chemotherapy				
HIPEC alone	Ref.		Ref.	
HIPEC plus EPIC^f^	0.67 (0.35, 1.26)	0.213	0.75 (0.44, 1.28)	0.291
Combinations of intraperitoneal chemotherapy-2				
5-fluorouracil/mitomycin C^g^	Ref.		Ref.	
Cisplatin only	0.70 (0.30, 1.61)	0.396	0.57 (0.29, 1.15)	0.116
Cisplatin plus another^h^	0.97 (0.50, 1.88)	0.918	0.88 (0.54, 1.44)	0.613
Cisplatin plus two others^i^	0.83 (0.11, 6.41)	0.861	0.95 (0.28, 3.21)	0.940
Histopathology				
Low-grade	Ref.		Ref.	
High-grade	1.80 (1.10, 2.93)	**0**.**019**	1.93 (1.15, 2.80)	**0**.**022**
Other major complications within 30 days^j^	0.98 (0.59, 1.64)	0.941	0.96 (0.63, 1.46)	0.837

*P* values in bold indicate <0.05.

ASA, American society of anesthesiologists; HIPEC, hyperthermia intraperitoneal chemotherapy; MAP, mean arterial pressure; EPIC, early postoperative intraperitoneal chemotherapy.

a Variables with *P* values <0.20 in univariable analyses were examined consecutively with multivariable analyses, applying a COX proportional hazard model. Completeness of cytoreduction score and peritoneal cancer index were not included because of correlation with complete cytoreduction. Artificial colloid or crystalloid transfusion, and estimated blood loss were not included because of correlation with duration of surgery. The increase of postoperative tumor markers was not included because of correlation with preoperative tumor markers increased.

b Represented function capacity of patients, ranged from 0 (total dependence) to 100 (complete independence), assessed using the 10-item scale (each item was scored with 0, 5, 10 and 15 points) by nurse at hospital admission (31).

c Prior surgical score (PSS) ranged from 0 to 3. PSS-0 was for no prior surgery or biopsy; PSS-1 was for surgery in one abdominal region; PSS-2 was for surgery in 2–5 regions; PSS-3 was for surgery in >5 regions.

d Completeness of cytoreduction (CC) score was used to record the volume of residual cancer, assessed after the surgical procedures. CC-0 signified no macroscopic residual disease remained; CC-1 signified no nodule greater than 2.5 mm in diameter remained; CC-2 signified nodule between 2.5 mm and 2.5 cm in diameter remained; and CC-3 signified nodule greater than 2.5 cm in diameter remained (32).

e Indicate areas under the threshold of MAP <65 mmHg, defined as sum of all areas below the given threshold, where each area was calculated with the use of trapezoid rule and linearly interpolating between measurements.

f Early postoperative intraperitoneal chemotherapy typically administered from postoperative days 2–6 as appropriate.

g Included 5-fluorouracil alone (4 cases), mitomycin C alone (1 case), and 5-fluorouracil + mitomycin C (142 cases).

h Included cisplatin plus 5-fluorouracil (171 cases), and cisplatin plus raltitrexed (2 case).

i Included cisplatin + 5-fluorouracil + raltitrexed (34 cases), cisplatin + 5-fluorouracil + mitomycin C (3 cases).

j Indicate Clavien-Dindo classification grade 3 or greater. Grade 3 indicated complications requiring radiological intervention, endoscopic, or surgical intervention with or without general anesthesia; grade 4 indicated complications requiring a return to the intensive care unit management, sepsis, and one or multiple organ failure; grade 5 indicated death within 30 days postoperative (34). Occurrence of acute kidney injury was not accounted for.

Sensitivity analyses in 294 cases with intraoperative temperature did not change our results. All raised tumor markers (HR = 10.32; 95% CI: 2.13, 50.02; *P* = 0.004), the CC-score (HR = 3.18–11.22; 95% CI: 1.12–29.41; *P* < 0.05), and high-grade PMP (HR = 2.72; 95% CI: 1.88, 3.75; *P* = 0.005) remained associated with OS; Barthel index (HR = 0.87; 95% CI: 0.74, 0.92; *P* = 0.042), preoperative 2 or 3 raised tumor markers (HR = 4.86–7.54; 95% CI: 1.45–24.97; *P* < 0.05), CC-score (HR = 3.42–4.77; 95% CI: 1.54–8.91; *P* < 0.05), and high-grade PMP (HR = 2.88; 95% CI: 1.52, 3.37; *P* = 0.004) remained associated with EFS; both intraoperative hypotension and intraoperative hyperthermia were not associated with OS or EFS (Supplementary Tables S1–S4).

## 4 Discussion

After CRS-HIPEC, preoperative tumor marker levels, Barthel index, CC-score, and PMP histology were independently associated with OS or EFS in patients with PMP from mucinous appendix neoplasms. The results may help improve the perioperative management of patients with PMP.

After a median follow-up of 40 months, the median OS was 56 (95% CI: 50, 61) months and the median EFS was 45 (95% CI: 40, 51) months in our study, shorter than other authors from China previous reported (12). That might be due to the different characteristics between the cohort patients: the PCI scores of our patients (75.7% of the patients with PCI ≥11) were much higher compared with the study from Yan et al. (52.8% of the patients with PCI >12) (12).

Of note, only 51.4% of our patients achieved complete cytoreduction (CC-0/1), substantially lower than the 66% to 93% reported by authors from other regions (36, 37). It was easily to be explained as following: firstly, a significant proportion (72.2% patients with PSS 0–2) of the patients came to our institution for CRS-HIPEC after unsuccessful previous surgical interventions; secondly, nearly half of the patients awaited professional CRS-HIPEC for more than 6 months from diagnosis; thirdly, factors such as higher PCI, male gender, and high pathological grade could also decrease the likelihood of complete cytoreduction (38).

Unsurprisingly, consistent with the other studies (7, 8, 10, 37, 39), our study also identified CC-score and PMP pathology associated with OS and EFS. Complete cytoreduction is probably the strongest prognostic factor in PMP by reducing the negative prognostic impact of histological grade (10, 38). And consistent with our result, many studies have showed that an increase in one or more of tumor markers was associated with poorer prognosis in PMP patients (13, 40).

In this study, a lower Barthel index weas found to be associated with a poorer EFS. Usually, a patient with lower Barthel index had a worse function status, with more comorbidities and a poorer health status, consequently more frequent postoperative complications (41) and even shorter survival (42).

93.8% of our patients experienced hypotension (MAP < 65 mmHg) during CRS-HIPEC, but it was not associated with OS and EFS in PMP patients. This raises concern regarding the special significance and innovation of this retrospective study. However, accumulating evidence showed that intraoperative blood pressure management was associated with long-term survival (23–26). Nearly all our patients experienced hypotension, which might limit the power of the statistical analyses. In addition, hypotension with different underlying pathophysiological causes might have different effects on organ perfusion and outcomes. Hemodynamic changes during HIPEC are complex due to heat stress, including increased cardiac output and heart rate, decreased stroke volume, MAP, and systemic vascular resistance (43). As we known, organ perfusion is proportional to the perfusion pressure and inversely proportional to the local vascular resistance. Depending on the relative extent of the changes in local vascular resistance compared to perfusion pressure, organ perfusion might remain stable or even increase, irrespective of decreased MAP, thereby leading to different effects on surgical outcomes (44). More studies are warranted to explore the relationship between intraoperative blood pressure management and long-term outcomes in PMP patients undergoing CRS-HIPEC.

The strength of this study was that the patients were from a single center, minimizing potential bias due to differences in local practice among hospitals. In addition, the study center is specialized in CRS-HIPEC. A French multicenter study of 301 patients with PMP showed that centers with less experience had poorer survival results (10). Still, this study also had limitations. The retrospective study design inherently limited data collection to information documented in medical records, and data such as primary tumor histology and prior chemotherapy were not recorded in detail. Furthermore, this study did not find significant associations between intraoperative hypotension/hyperthermia and OS/EFS. Notably, important factors including cardiac output, vascular resistance, and intraoperative hypotension interventions were not be analyzed. Although it was declared as a strength, the single-center nature of the study also limits generalizability because of the local protocols. Because CRS-HIPEC for PMP is standard in our center, no contemporary control group could be included. These shortcomings highlighted the critical need for multicentric studies with larger size and control groups to corroborate and broaden the insights into the prognostic factors for both short- and long-term outcomes in PMP.

## 5 Conclusions

After CRS-HIPEC, preoperative tumor marker levels, Barthel Index, CC-score, and PMP histology were independently associated with OS or EFS; intraoperative hypotension and hyperthermia were not associated with both OS and EFS.

## Data Availability

The original contributions presented in the study are included in the article/Supplementary Material, further inquiries can be directed to the corresponding author.

## Supplementary material

Supplementary Table S1 Baseline data in patients with intraoperative temperature and their association with long-term survival (univariable COX regression analyses)

Supplementary Table S2 Perioperative data in patients with intraoperative temperature and their association with long-term survival (univariable COX regression analyses)

Supplementary Table S3 Long-term follow-up results in patients with intraoperative temperature

Supplementary Table S4 Predictors of overall and event-free survival in patients with intraoperative temperature (multivariable COX proportional hazard model)

Supplementary Table S5 Characteristics and clinical data between CC-0/1 and CC-2/3
