# Supplementary material for: Long-term outcomes of pseudomyxoma peritonei after cytoreductive surgery and hyperthermic intraperitoneal chemotherapy and its relevant risk factors in China: a retrospective study
Source: Front Surg. 2026 Jan 30;13:1692847. doi: 10.3389/fsurg.2026.1692847 (PMC12901328; doi:10.3389/fsurg.2026.1692847)
Supplement: Supplementary Table S1 — Baseline data in patients with intraoperative temperature and their association with long-term survival (univariable COX regression analyses) [file Supplementaryfile1.docx]

Table S1. Baseline data in patients with intraoperative temperature and their association with long-term survival (univariable COX regression analyses)

| Characteristics | Statistical description  (n=294) | Overall survival | | | | Event-free survival | |
| --- | --- | --- | --- | --- | --- | --- | --- |
|  |  | Hazard ratio (95% CI) | *P* value | | Hazard ratio (95% CI) | | *P* value |
| Demographic characteristics |  |  | |  | |  |  |
| Age, year | 57 ± 10 | 1.01 (0.99, 1.03) | | 0.177 | | 1.00 (0.98, 1.02) | 0.855 |
| Female sex | 189 (64.3%) | 1.20 (0.77, 1.88) | | 0.420 | | 1.06 (0.73, 1.54) | 0.747 |
| Body mass index, kg/m^2^ | 23.2 (21.3, 25.9) | 0.96 (0.89, 1.02) | | 0.207 | | 1.00 (0.94, 1.05) | 0.897 |
| General status |  |  | |  | |  |  |
| Charlson comorbidity index ^a^ | 8.0 (8.0, 8.0) | 0.94 (0.63, 1.41) | | 0.767 | | 1.07 (0.79, 1.44) | 0.674 |
| ASA physical status ^b^ |  |  | |  | |  |  |
| I-II | 149 (50.7%) | Ref. | |  | | Ref. |  |
| III-IV | 145 (49.3%) | 1.49 (0.97, 2.29) | | 0.071 | | 1.31 (0.91, 1.88) | 0.148 |
| Barthel index ^c^, per 10-point increase | 10.0 (9.0, 10.0) | 0.87 (0.75, 1.00) | | 0.047 | | 0.85 (0.75, 0.97) | 0.013 |
| History of previous therapy |  |  | |  | |  |  |
| Prior surgical score ^d^ |  |  | |  | |  |  |
| 0-2 | 215 (73.1%) | Ref. | |  | | Ref. |  |
| 3 | 79 (26.9%) | 1.42 (0.89, 2.25) | | 0.139 | | 1.66 (1.14, 2.43) | 0.009 |
| Prior chemotherapy | 61 (20.7%) | 1.31 (0.81, 2.11) | | 0.273 | | 1.59 (1.07, 2.37) | 0.022 |
| Prior HIPEC exposure | 63 (21.4%) | 1.40 (0.84, 2.35) | | 0.198 | | 1.42 (0.93, 2.16) | 0.105 |
| Preoperative laboratory tests |  |  | |  | |  |  |
| Hemoglobin, g/L | 115 ± 17 [8] | 0.99 (0.97, 1.00) | | 0.024 | | 0.99 (0.98, 1.00) | 0.007 |
| Albumin, g/L | 36 ± 4 [3] | 0.95 (0.91, 1.00) | | 0.055 | | 0.93 (0.89, 0.97) | <0.001 |
| Tumor markers (CA125, CA199, CEA) |  |  | |  | |  |  |
| Normal | 62 (21.1%) | Ref. | |  | | Ref. |  |
| 1 raised | 88 (29.9%) | 6.01 (1.42, 25.46) | | 0.015 | | 4.41 (1.57, 12.41) | 0.005 |
| 2 raised | 58 (19.7%) | 9.71 (2.24, 42.07) | | 0.002 | | 8.85 (3.12, 25.11) | <0.001 |
| All raised | 86 (29.3%) | 19.72 (4.75, 81.77) | | <0.001 | | 11.76 (4.24, 32.66) | <0.001 |

Data are mean ± SD, n (%), or median (interquartile range). Numbers in square brackets indicate patients with missing data. *P* values in bold indicate <0.20.

ASA, American society of anesthesiologists; HIPEC, hyperthermia intraperitoneal chemotherapy.

^a^ Assessed according to Charlson comorbidity index (12 items).

^b^ Included ASA I (2 cases), ASA II (147 cases), ASA III (140 cases), and ASA IV (5 cases).

^c^ Represented function capacity of patients, ranged from 0 (total dependence) to 100 (complete independence), assessed using the 10-item scale (each item was scored with 0, 5, 10 and 15 points) by nurse at hospital admission.

^d^ Prior surgical score (PSS) ranged from 0-3. PSS-0 was for no prior surgery or biopsy; PSS-1 was for surgery in one abdominal region; PSS-2 was for surgery in 2-5 regions; PSS-3 was for surgery in >5 regions.

Table S2. Perioperative data in patients with intraoperative temperature and their association with long-term survival (univariable COX regression analyses)

| Characteristics | Statistical description (n=294) | Overall survival | | | | | Event-free survival | | |
| --- | --- | --- | --- | --- | --- | --- | --- | --- | --- |
|  |  | Hazard Ratio (95% CI) | | *P* value | | Hazard Ratio (95% CI) | | *P* value | |
| Intraoperative data |  | |  | |  | |  |  | |
| Peritoneal cancer index ^a^ |  | |  | |  | |  | |  |
| 0-10 | 57 (19.4%) | | Ref. | |  | | Ref. | |  |
| 11-20 | 39 (13.3%) | | 5.17 (1.66, 16.09) | | 0.005 | | 4.14 (1.70, 10.09) | | 0.002 |
| 21-30 | 116 (39.5%) | | 5.90 (2.12, 16.41) | | <0.001 | | 4.90 (2.24, 10.71) | | <0.001 |
| 31-39 | 82 (27.9%) | | 7.60 (2.60, 22.23) | | <0.001 | | 5.67 (2.50, 12.88) | | <0.001 |
| Completeness of cytoreduction ^b^ |  | |  | |  | |  | |  |
| 0 | 55 (18.7%) | | Ref. | |  | | Ref. | |  |
| 1 | 75 (25.5%) | | 4.75 (1.79, 12.61) | | 0.002 | | 2.90 (1.40, 6.02) | | 0.004 |
| 2 | 92 (31.3%) | | 4.98 (1.96, 12.66) | | <0.001 | | 3.52 (1.79, 6.91) | | <0.001 |
| 3 | 72 (24.5%) | | 6.81 (2.55, 18.23) | | <0.001 | | 4.53 (2.18, 9.40) | | <0.001 |
| Complete cytoreduction ^c^ | 130 (44.2%) | | 0.51 (0.32, 0.81) | | 0.004 | | 0.50 (0.34, 0.75) | | <0.001 |
| Duration of surgery, h | 7.7 (6.5, 9.6) | | 1.10 (0.99, 1.22) | | 0.078 | | 1.08 (1.00, 1.18) | | 0.063 |
| Artificial colloid, per 500 mL | 5.2 (4.0, 6.3) | | 1.19 (1.07, 1.32) | | 0.002 | | 1.15 (1.05, 1.26) | | 0.004 |
| Crystalloid, per 500 mL | 6.6 (5.4, 8.4) | | 1.17 (1.05, 1.30) | | 0.005 | | 1.12 (1.03, 1.23) | | 0.012 |
| Blood transfusion | 178 (60.5%) | | 2.43 (1.39, 4.25) | | 0.002 | | 2.26 (1.44, 3.54) | | <0.001 |
| Estimated blood loss, per 500 mL | 3.0 (1.4, 3.2) | | 1.21 (1.11, 1.33) | | <0.001 | | 1.16 (1.08, 1.26) | | <0.001 |
| Area under threshold of MAP ^d^, 30 mmHg×min |  | |  | |  | |  | |  |
| <75 mmHg | 86.3 (44.5, 139.0) | | 1.00 (1.00, 1.01) | | 0.053 | | 1.00 (1.00, 1.01) | | 0.014 |
| <70 mmHg | 41.8 (17.9, 76.6) | | 1.00 (1.00, 1.01) | | 0.050 | | 1.01 (1.00, 1.01) | | 0.013 |
| <65 mmHg | 13.9 (3.9, 31.5) | | 1.01 (1.00, 1.01) | | 0.047 | | 1.01 (1.00, 1.01) | | 0.018 |
| Area above threshold of temperature, °C×min |  | |  | |  | |  | |  |
| >38.0°C | 4.0 (0.0, 39.0) | | 1.00 (1.00, 1.01) | | 0.739 | | 1.00 (1.00, 1.01) | | 0.851 |
| >37.5°C | 33.8 (0.97, 90.6) | | 1.00 (1.00, 1.00) | | 0.575 | | 1.00 (1.00, 1.00) | | 0.990 |
| >37.0°C | 82.7 (31.9, 160.6) | | 1.00 (1.00, 1.00) | | 0.556 | | 1.00 (1.00, 1.00) | | 0.977 |
| Intraperitoneal chemotherapy regimens |  | |  | |  | |  | |  |
| Intraperitoneal chemotherapy |  | |  | |  | |  | |  |
| HIPEC alone | 75 (25.5%) | | Ref. | |  | | Ref. | |  |
| HIPEC plus EPIC ^e^ | 219 (74.5%) | | 0.48 (0.31, 0.76) | | 0.002 | | 0.62 (0.42, 0.91) | | 0.015 |
| Combinations of intraperitoneal chemotherapy-1 |  | |  | |  | |  | |  |
| Non-cisplatin containing | 138 (46.9%) | | Ref. | |  | | Ref. | |  |
| Cisplatin containing | 156 (53.1%) | | 1.05 (0.63, 1.73) | | 0.863 | | 1.02 (0.69, 1.52) | | 0.905 |
| Combinations of intraperitoneal chemotherapy-2 |  | |  | |  | |  | |  |
| 5-fluorouracil/mitomycin C ^f^ | 138 (46.9%) | | Ref. | |  | | Ref. | |  |
| Cisplatin only | 44 (15.0%) | | 1.44 (0.70, 2.96) | | 0.324 | | 1.18 (0.65, 2.14) | | 0.591 |
| Cisplatin plus another ^g^ | 82 (27.9%) | | 0.94 (0.51, 1.73) | | 0.835 | | 0.99 (0.62, 1.58) | | 0.968 |
| Cisplatin plus two others ^h^ | 30 (10.2%) | | 0.55 (0.75, 4.00) | | 0.554 | | 0.79 (0.25, 2.54) | | 0.697 |
| Postoperative data |  | |  | |  | |  | |  |
| Histopathology subtype |  | |  | |  | |  | |  |
| Low-grade | 210 (71.4%) | | Ref. | |  | | Ref. | |  |
| High-grade | 84 (28.6%) | | 2.04 (1.33, 3.12) | | 0.001 | | 1.53 (1.05, 2.23) | | 0.029 |
| Tumor markers (CA125, CA199, CEA) |  | |  | |  | |  | |  |
| Normal | 119 (40.5%) | | Ref. | |  | | Ref. | |  |
| 1 raised | 82 (27.9%) | | 1.59 (0.90, 2.81) | | 0.110 | | 1.70 (1.05, 2.77) | | 0.033 |
| ≥2 raised ^i^ | 93 (31.6%) | | 3.43 (2.02, 5.83) | | <0.001 | | 3.43 (2.19, 5.38) | | <0.001 |
| Acute kidney injury ^j^ | 24 (8.2%) | | 1.53 (0.71, 3.33) | | 0.280 | | 1.69 (0.90, 3.15) | | 0.101 |
| Other major complications within 30 days ^k^ | 77 (26.2%) | | 1.18 (0.74, 1.92) | | 0.480 | | 1.05 (0.70, 1.58) | | 0.809 |

Data are n (%), or median (interquartile range). *P* values in bold indicate <0.20.

MAP, mean artery pressure; HIPEC, hyperthermia intraperitoneal chemotherapy; EPIC, early postoperative intraperitoneal chemotherapy.

^a^ Peritoneal cancer index quantified peritoneal disease burden, and comprised a score of 0-3 in 13 abdominopelvic regions to a computed index ranging from 0-39. It was determined intraoperatively after abdominopelvic cavities exposure, but before any peritonectomy procedures performed.

^b^ Completeness of cytoreduction (CC) score was used to record the volume of residual cancer, assessed after the surgical procedures. CC-0 signified no macroscopic residual disease remained; CC-1 signified no nodule greater than 2.5 mm in diameter remained; CC-2 signified nodule between 2.5 mm and 2.5 cm in diameter remained; and CC-3 signified nodule greater than 2.5 cm in diameter remained.

^c^ A state of total macroscopic disease eradiation, encompasses CC-0 (no macroscopic residual disease remained) as well as CC-1 (no nodule greater than 2.5 mm in diameter remained).

^d^ Indicate areas under the specialized MAP thresholds, defined as sum of all areas below the given threshold, where each area was calculated with the use of trapezoid rule and linearly interpolating between measurements.

^e^ Early postoperative intraperitoneal chemotherapy typically administered from postoperative days 2-6 as appropriate.

^f^ Included 5-fluorouracil alone (4 cases), mitomycin C alone (1 case), and 5-fluorouracil+mitomycin C (133 cases).

^g^ Included cisplatin plus 5-fluorouracil (81 cases), and cisplatin plus raltitrexed (1 case).

^h^ Included cisplatin + 5-fluorouracil + raltitrexed (27 cases), cisplatin + 5-fluorouracil + mitomycin C (3 cases).

^i^ Typically measured 14 days after surgery. If discharged within 14 days the last value was recorded.

^j^ Defined either serum creatinine increased by ≥0.3 mg/dL within 48 hours, or increased to ≥1.5 times baseline within the previous 7 days postoperative according to Kidney Disease Improving Global Outcome (KDIGO) criteria.

^k^ Indicate Clavien-Dindo classification grade 3 or greater. Grade 3 indicated complications requiring radiological intervention, endoscopic, or surgical intervention with or without general anesthesia; grade 4 indicated complications requiring a return to the intensive care unit management, sepsis, and one or multiple organ failure; grade 5 indicated death within 30 days postoperative. Occurrence of acute kidney injury was not accounted for.

Table S3. Long-term follow-up results in patients with intraoperative temperature

| Characteristics | Statistical description (n=294) |
| --- | --- |
| Duration of follow-up, month | 40 (21, 60) |
| Re-hospitalization for PMP ^a^ | 94 (32.0%) |
| Scheduled re-hospitalization ^b^ | 167 (56.8%) |
| Number of deaths during follow-up | 86 (29.3%) |
| Number of events during follow-up ^c^ | 120 (40.8%) |
| Event-free survival, month ^d^ | 42 (33, 51) |
| Overall survival, month (all-cause death) ^e^ | 54 (51, 58) |

Data are n (%), median (interquartile range), or median (95% confidence interval).

PMP, pseudomyxoma peritonei; HIPEC, hyperthermia intraperitoneal chemotherapy.

^a^ Re-hospitalization for intravenous chemotherapy, HIPEC, or redo-cytoreduction surgery.

^b^ Re-hospitalization for intravenous chemotherapy, HIPEC, regular follow-up visits, or removal of ureteral stents.

^c^ Events refers to PMP recurrence (for patients achieved CC-0/1) or progression (for patients achieved CC-2/3), unplanned re-hospitalization for non-PMP serious diseases (included pyelostomy, inferior vena cava filter placement, endoscopic surgery, and surgery for hernia or other cancers), or all-cause death, whichever occurred.

^d^ Defined as time interval from surgery to PMP recurrence/progression, unplanned re-hospitalization for non-PMP serious disease, or all-cause death, whichever occurred first.

^e^ Defined as time interval from surgery to all-cause death.

Table S4. Predictors of overall and event-free survival in patients with intraoperative temperature (multivariable COX proportional hazard model) ^a^

| Characteristics | Overall survival | | Event-free survival | |
| --- | --- | --- | --- | --- |
|  | Hazard Ratio (95% CI) | *P* value | Hazard Ratio (95% CI) | *P* value |
| Age, year | 1.00 (0.98, 1.03) | 0.713 | 0.98 (0.96, 1.00) | 0.081 |
| ASA physical status |  |  |  |  |
| I-II | Ref. |  | Ref. |  |
| III-IV | 1.33 (0.80, 2.23) | 0.276 | 1.15 (0.75, 1.77) | 0.527 |
| Barthel index ^b^, per 10-point increase | 0.91 (0.78, 1.03) | 0.073 | 0.87 (0.74, 0.92) | 0.042 |
| Prior surgical score ^c^ |  |  |  |  |
| 0-2 | Ref. |  | Ref. |  |
| 3 | 0.90 (0.50, 1.60) | 0.709 | 1.27 (0.80, 2.01) | 0.313 |
| Prior chemotherapy | 1.04 (0.59, 1.84) | 0.893 | 1.11 (0.69, 1.76) | 0.671 |
| Prior HIPEC exposure | 1.07 (0.58, 1.96) | 0.840 | 1.18 (0.72, 1.94) | 0.509 |
| Preoperative hemoglobin, g/L | 0.99 (0.97, 1.01) | 0.390 | 1.00 (0.99, 1.02) | 0.864 |
| Preoperative albumin, g/L | 1.04 (0.96, 1.12) | 0.349 | 0.97 (0.91, 1.03) | 0.271 |
| Preoperative tumor markers (CA125, CA199, CEA) |  |  |  |  |
| Normal | Ref. |  | Ref. |  |
| 1 raised | 3.95 (0.87, 17.88) | 0.075 | 3.05 (0.99, 9.36) | 0.052 |
| 2 raised | 4.86 (0.97, 24.44) | 0.055 | 4.86 (1.45, 16.30) | 0.011 |
| All raised | 10.32 (2.13, 50.02) | 0.004 | 7.54 (2.28, 24.97) | <0.001 |
| Completeness of cytoreduction ^d^ |  |  |  |  |
| 0 | Ref. |  | Ref. |  |
| 1 | 3.18 (1.12, 11.08) | 0.016 | 2.23 (1.00, 5.34) | 0.058 |
| 2 | 6.67 (2.00, 15.57) | 0.007 | 3.42 (1.54, 7.44) | 0.011 |
| 3 | 11.22 (4.27, 29.41) | 0.001 | 4.77 (2.26, 8.91) | 0.004 |
| Duration of surgery, h | 0.96 (0.83, 1.12) | 0.612 | 0.93 (0.82, 1.05) | 0.247 |
| Blood transfusion | 0.83 (0.38, 1.84) | 0.651 | 1.08 (0.57, 2.05) | 0.806 |
| Aera under MAP <65 mmHg ^e^, 30 mmHg×min | 1.00 (0.99, 1.01) | 0.639 | 1.00 (1.00, 1.01) | 0.718 |
| Area above temperature >38°C, °C×min | 1.00 (0.99, 1.01) | 0.588 | 1.00 (1.00, 1.01) | 0.161 |
| Intraperitoneal chemotherapy |  |  |  |  |
| HIPEC alone | Ref. |  | Ref. |  |
| HIPEC plus EPIC ^f^ | 0.76 (0.39, 1.49) | 0.424 | 0.82 (0.47, 1.44) | 0.493 |
| Combinations of intraperitoneal chemotherapy-2 |  |  |  |  |
| 5-fluorouracil/mitomycin C ^g^ | Ref. |  | Ref. |  |
| Cisplatin only | 0.97 (0.36, 2.66) | 0.958 | 0.85 (0.38, 1.92) | 0.701 |
| Cisplatin plus another ^h^ | 1.39 (0.67, 2.89) | 0.383 | 1.22 (0.71, 2.10) | 0.471 |
| Cisplatin plus two others ^i^ | 0.72 (0.09, 5.67) | 0.754 | 0.83 (0.24, 2.91) | 0.768 |
| Histopathology |  |  |  |  |
| Low-grade | Ref. |  | Ref. |  |
| High-grade | 2.72 (1.88, 3.75) | 0.005 | 2.88 (1.52, 3.37) | 0.004 |
| Acute kidney injury ^j^ | 1.36 (0.51, 3.59) | 0.538 | 1.70 (0.79, 3.65) | 0.175 |

*P* values in bold indicate <0.05.

ASA, American society of anesthesiologists; HIPEC, hyperthermia intraperitoneal chemotherapy; MAP, mean arterial pressure; EPIC, early postoperative intraperitoneal chemotherapy.

^a^ Variables with *P* values <0.20 in univariable analyses and factors considered clinically significant were examined consecutively with multivariable analyses, applying a COX proportional hazard model. Complete cytoreduction and peritoneal cancer index were not included because of correlation with completeness of cytoreduction score. Artificial colloid or crystalloid transfusion, and estimated blood loss were not included because of correlation with duration of surgery. The increase of postoperative tumor markers was not included because of correlation with preoperative tumor markers increased.

^b^ Represented function capacity of patients, ranged from 0 (total dependence) to 100 (complete independence), assessed using the 10-item scale (each item was scored with 0, 5, 10 and 15 points) by nurse at hospital admission.

^c^ Prior surgical score (PSS) ranged from 0-3. PSS-0 was for no prior surgery or biopsy; PSS-1 was for surgery in one abdominal region; PSS-2 was for surgery in 2-5 regions; PSS-3 was for surgery in >5 regions.

^d^ Completeness of cytoreduction (CC) score was used to record the volume of residual cancer, assessed after the surgical procedures. CC-0 signified no macroscopic residual disease remained; CC-1 signified no nodule greater than 2.5 mm in diameter remained; CC-2 signified nodule between 2.5 mm and 2.5 cm in diameter remained; and CC-3 signified nodule greater than 2.5 cm in diameter remained.

^e^ Indicate areas under the threshold of MAP <65 mmHg, defined as sum of all areas below the given threshold, where each area was calculated with the use of trapezoid rule and linearly interpolating between measurements.

^f^ Early postoperative intraperitoneal chemotherapy typically administered from postoperative days 2-6 as appropriate.

^g^ Included 5-fluorouracil alone (4 cases), mitomycin C alone (1 case), and 5-fluorouracil+mitomycin C (133 cases).

^h^ Included cisplatin plus 5-fluorouracil (81 cases), and cisplatin plus raltitrexed (1 case).

^i^ Included cisplatin + 5-fluorouracil + raltitrexed (27 cases), cisplatin + 5-fluorouracil + mitomycin C (3 cases).

^j^ Defined either serum creatinine increased by ≥0.3 mg/dL within 48 hours, or increased to ≥1.5 times baseline within the previous 7 days postoperative according to Kidney Disease Improving Global Outcome (KDIGO) criteria.

Table S5. Characteristics and clinical data between CC-0/1 and CC-2/3

| Characteristics | CCR 0/1 | CCR 2/3 | *P* value |
| --- | --- | --- | --- |
| Demographic characteristics |  |  |  |
| Age, year | 56 ± 11 | 58 ± 10 | 0.004 |
| Female sex | 153 (68.9%) | 128 (61.0%) | 0.102 |
| Body mass index, kg/m^2^ | 23.9 (21.5, 26.5) | 22.8 (20.8, 25.7) | 0.015 |
| General status |  |  |  |
| Charlson Comorbidity Index ^a^ | 8.0 (8.0, 8.0) | 8.0 (8.0, 8.0) |  |
| ASA physical status |  |  | <0.001 |
| I-II | 155 (69.8%) | 86 (41.0%) |  |
| III-IV | 67 (30.2%) | 124 (59.0%) |  |
| Barthel index ^b^, per 10-point increase | 10.0 (9.5, 10.0) | 10.0 (9.0, 10.0) | 0.005 |
| History of previous therapy |  |  |  |
| Prior surgical score ^c^ |  |  | 0.005 |
| 0-2 | 174 (78.4%) | 138 (65.7%) |  |
| 3 | 48 (21.6%) | 72 (34.3%) |  |
| Prior chemotherapy | 30 (13.5%) | 53 (25.2%) | 0.003 |
| Prior HIPEC exposure | 36 (16.2%) | 52 (24.8%) | 0.037 |
| Preoperative laboratory tests |  |  |  |
| Hemoglobin, g/L | 123 ± 16 [6] | 111 ± 16 [8] | <0.001 |
| Albumin, g/L | 39 ± 4 [7] | 35 ± 4 [3] | <0.001 |
| Tumor markers (CA125, CA199, CEA) |  |  | <0.001 |
| Normal | 94 (42.3%) | 12 (5.7%) |  |
| 1 raised | 60 (27.0%) | 53 (25.2%) |  |
| 2 raised | 37 (16.7%) | 61 (29.0%) |  |
| All raised | 31 (14.0%) | 84 (40.0%) |  |
| Intraoperative data |  |  |  |
| Peritoneal cancer index ^d^ |  |  | <0.001 |
| 0-10 | 104 (46.8%) | 1 (0.5%) |  |
| 11-20 | 47 (21.2%) | 11 (5.2%) |  |
| 21-30 | 60 (27.0%) | 93 (44.3%) |  |
| 31-39 | 11 (5.0%) | 105 (50.0%) |  |
| Duration of surgery, h | 7.7 (6.2, 9.7) | 8.2 (6.8,9.6) | 0.058 |
| Artificial colloid, per 500 mL | 4.4 (3.2, 6.2) | 5.4 (4.4, 6.8) | <0.001 |
| Crystalloid, per 500 mL | 6.4 (4.6, 8.2) | 7.2 (6.0, 8.9) | <0.001 |
| Blood transfusion | 86 (38.7%) | 158 (75.2%) | <0.001 |
| Estimated blood loss, per 500 mL | 1.4 (0.6, 3.0) | 3.0 (2.0, 4.0) | <0.001 |
| Area under threshold of MAP ^e^, per 30 mmHg×min |  |  |  |
| <75 mmHg | 78 (35, 123) | 109 (61, 155) | <0.001 |
| <70 mmHg | 34 (11, 64) | 54 (25, 87) | <0.001 |
| <65 mmHg | 11 (2, 25) | 20 (7, 38) | <0.001 |
| Intraperitoneal chemotherapy regimens |  |  |  |
| Intraperitoneal chemotherapy |  |  | <0.001 |
| HIPEC alone | 38 (17.1%) | 68 (32.4%) |  |
| HIPEC plus EPIC ^f^ | 184 (82.9%) | 142 (67.6%) |  |
| Combinations of intraperitoneal chemotherapy-1 |  |  | <0.001 |
| Non-cisplatin containing | 56 (25.2%) | 90 (42.9%) |  |
| Cisplatin containing | 166 (74.8%) | 120 (57.1%) |  |
| Combinations of intraperitoneal chemotherapy-2 |  |  | <0.001 |
| 5-fluorouracil/mitomycin C | 57 (25.7%) | 90 (42.9%) |  |
| Cisplatin only | 35 (15.8%) | 40 (19.0%) |  |
| Cisplatin plus another | 110 (49.5%) | 63 (30.0%) |  |
| Cisplatin plus two others | 20 (9.0%) | 17 (8.1%) |  |
| Postoperative data |  |  |  |
| Histopathology subtype |  |  | 0.914 |
| Low-grade | 165 (74.3%) | 158 (75.2%) |  |
| High-grade | 57 (25.7%) | 52 (24.8%) |  |
| Tumor markers (CA125, CA199, CEA) ^g^ |  |  | <0.001 |
| Normal | 153 (68.9%) | 45 (21.4%) |  |
| 1 raised | 44 (19.8%) | 65 (31.0%) |  |
| ≥2 raised | 25 (11.3%) | 100 (47.6%) |  |
| Acute kidney injury ^h^ | 20 (9.0%) | 23 (11.0%) | 0.608 |
| Other major complications within 30 days ^i^ | 45 (20.3%) | 48 (22.9%) | 0.591 |
| Long-term outcomes |  |  |  |
| Duration of follow-up, month | 41 (27, 55) | 38 (22, 54) | 0.086 |
| Re-hospitalization for PMP ^j^ | 64 (28.8%) | 65 (31.0%) | 0.706 |
| Scheduled re-hospitalization ^k^ | 132 (59.5%) | 115 (54.8%) | 0.374 |
| Number of deaths during follow-up | 34 (15.3%) | 60 (28.6%) | 0.001 |
| Number of events during follow-up ^l^ | 53 (23.9%) | 87 (41.4%) | <0.001 |
| Event-free survival, month ^m^ | 62 (95% CI 42-81) | 39 (95% CI 30-49) | 0.013 * |
| Overall survival, month (all-cause death) ^n^ | 67 (95% CI 60-75) | 52 (95% CI 46-58) | 0.002 * |

Data are mean ± SD, n (%), or median (interquartile range). Numbers in square brackets indicate patients with missing data. *P* values in bold indicate <0.05. *P* values with * were from log-rank tests.

CC, Completeness of cytoreduction; ASA, American society of anesthesiologists; HIPEC, hyperthermia intraperitoneal chemotherapy; MAP, mean artery pressure; EPIC, early postoperative intraperitoneal chemotherapy; PMP, pseudomyxoma peritonei.

^a^ Assessed according to Charlson comorbidity index (12 items) (18).

^b^ Represented function capacity of patients, ranged from 0 (total dependence) to 100 (complete independence), assessed using the 10-item scale (each item was scored with 0, 5, 10 and 15 points) by nurse at hospital admission (20).

^c^ Prior surgical score (PSS) ranged from 0-3. PSS-0 was for no prior surgery or biopsy; PSS-1 was for surgery in one abdominal region; PSS-2 was for surgery in 2-5 regions; PSS-3 was for surgery in >5 regions.

^d^ Peritoneal cancer index quantified peritoneal disease burden, and comprised a score of 0-3 in 13 abdominopelvic regions to a computed index ranging from 0-39. It was determined intraoperatively after abdominopelvic cavities exposure, but before any peritonectomy procedures performed (21).

^e^ Indicate areas under the specialized MAP thresholds, defined as sum of all areas below the given threshold, where each area was calculated with the use of trapezoid rule and linearly interpolating between measurements.

^f^ Early postoperative intraperitoneal chemotherapy typically administered from postoperative days 2-6 as appropriate.

^g^ Typically measured 14 days after surgery. If discharged within 14 days the last value was recorded.

^h^ Defined either serum creatinine increased by ≥0.3 mg/dL within 48 hours, or increased to ≥1.5 times baseline within the previous 7 days postoperative according to Kidney Disease Improving Global Outcome (KDIGO) criteria (22).

^i^ Indicate Clavien-Dindo classification grade 3 or greater. Grade 3 indicated complications requiring radiological intervention, endoscopic, or surgical intervention with or without general anesthesia; grade 4 indicated complications requiring a return to the intensive care unit management, sepsis, and one or multiple organ failure; grade 5 indicated death within 30 days postoperative. Occurrence of acute kidney injury was not accounted for (23).

^j^ Re-hospitalization for intravenous chemotherapy, HIPEC, or redo-cytoreduction surgery.

^k^ Re-hospitalization for intravenous chemotherapy, HIPEC, regular follow-up visits, or removal of ureteral stents.

^l^ Events refers to PMP recurrence (for patients achieved CC-0/1) or progression (for patients achieved CC-2/3), unplanned re-hospitalization for non-PMP serious diseases (included pyelostomy, inferior vena cava filter placement, endoscopic surgery, and surgery for hernia or other cancers), or all-cause death, whichever occurred.

^m^ Defined as time interval from surgery to PMP recurrence/progression, unplanned re-hospitalization for non-PMP serious disease, or all-cause death, whichever occurred first.

^n^ Defined as time interval from surgery to all-cause death.
